# Naive Bayes-Guided Bat Algorithm for Feature Selection

**DOI:** 10.1155/2013/325973

**Published:** 2013-12-14

**Authors:** Ahmed Majid Taha, Aida Mustapha, Soong-Der Chen

**Affiliations:** ^1^College of Graduate Studies, Universiti Tenaga Nasional, 43000 Kajang, Selangor, Malaysia; ^2^Faculty of Computer Science and Information Technology, Universiti Putra Malaysia, 43400 Serdang, Selangor, Malaysia; ^3^College of Information Technology, Universiti Tenaga Nasional, 43000 Kajang, Selangor, Malaysia

## Abstract

When the amount of data and information is said to double in every 20 months or so, feature selection has become highly important and beneficial. Further improvements in feature selection will positively affect a wide array of applications in fields such as pattern recognition, machine learning, or signal processing. Bio-inspired method called Bat Algorithm hybridized with a Naive Bayes classifier has been presented in this work. The performance of the proposed feature selection algorithm was investigated using twelve benchmark datasets from different domains and was compared to three other well-known feature selection algorithms. Discussion focused on four perspectives: number of features, classification accuracy, stability, and feature generalization. The results showed that BANB significantly outperformed other algorithms in selecting lower number of features, hence removing irrelevant, redundant, or noisy features while maintaining the classification accuracy. BANB is also proven to be more stable than other methods and is capable of producing more general feature subsets.

## 1. Introduction

The motivations to perform feature selection in a classification experiment are two-fold. The first is to enhance the classifier performance by selecting only useful features and removing irrelevant, redundant, or noisy features. The second is to reduce the number of features when classification algorithms could not scale up to the size of feature set either in time or space. In general, feature selection consists of two essential steps, which are searching for desired subset using some search strategies and evaluating the subset produced. A search strategy in searching the feature subset could be exhaustive or approximate. While exhaustive search strategy evaluates all probabilities of the feature subset, approximate search strategy only generates high quality solutions with no guarantee of finding a global optimal solution [1].

One of the most prominent algorithms in exhaustive search is branch and bound method [2]. Exhaustive search guarantees optimal solution but this method is not practical for even a medium-sized dataset as finding the optimal subset of features is an NP-hard problem [3]. For  *N*  number of features, the number of possible solutions will be exponential to 2^*N*^. Since exhaustive search is not practical, research effort and focus on search strategies have since shifted to metaheuristic algorithms, which are considered as a subclass of approximate methods. The literature has shown that the metaheuristic algorithms are capable of handling large-size problem instances with satisfactory solutions within a reasonable time [4–7].

After searching for feature subset, each candidate from the resulting subset generated needs to be evaluated based on some predetermined assessment criteria. There are three categories of feature subset evaluations depending on how the searching strategy is being associated with the classification model, whether as filter, wrapper, or embedded methods. These three categories will be explained in more detail in the next section.

Nonetheless, the main challenge in feature selection is to select the minimal subset of features with modicum or no loss of classification accuracy. While the literature has shown numerous developments in achieving this [8–10], the basis of comparison is rather limited in terms of number of features, classification accuracy, stability, or feature generalization in isolation. Generalization of the produced features is important to investigate their effect on the performance of different classifiers.

In view of this, the objectives of this paper are as follows: first to design a new hybrid algorithm that exploits a Naive Bayes algorithm to guide a Bat Algorithm, second to evaluate the performance of the proposed hybrid algorithm against other well-known feature selection algorithms, and third to test the effect of the resulting features in terms of generalization using three different classifiers. The remainder of this paper is organized as follows.  Section 2 reviews the related works on searching and evaluating algorithms in feature selection.  Section 3 details out the principles of Naive Bayes algorithms, Section 4 presents mechanics of the Bat Algorithm, and Section 5 introduces the proposed Naïve Bayes-guided Bat Algorithm for feature selection. Next, Section 6 describes the experimental settings, Section 7 discusses implications of the results, and, finally, Section 8 concludes with some recommendations for future work.

## 2. Related Work

The application of metaheuristics algorithms in searching the feature subset has shown high effectiveness as well as efficiency to solve complex and large problems in feature selection. In general, there are two categories of metaheuristic search algorithms: single solution-based metaheuristics (SBM) that manipulate and transform a single solution during the search and population-based metaheuristics (PBM) where a whole population of solutions is evolved. The simplest and oldest SBM method used in feature selection is Hill Climbing (HC) algorithm [1, 11]. This algorithm starts with a random initial solution and swaps the current solution with a neighboring solution in the following iteration in order to improve the quality of solution. Searching will stop only when all the neighboring candidate subsets are poorer than the current solution, which means that the algorithm will be most probably trapped in local optimum [4].

In order to overcome this problem, Simulated Annealing (SA) is proposed [10]. SA accepts the worse moves that commensurate to the parameter determined at the initial stage, called the temperature, which is inversely proportional to the change of the fitness function. In more recent work, a modified SA algorithm called the Great Deluge Algorithm (GDA) is proposed [12] to provide a deterministic acceptance function of the neighboring solutions. Tabu Search (TS) also accepts nonimproving solutions to escape from local optima. TS stores information related to the search process, which is a list of all previous solutions or moves in what is termed as Tabu list [13, 14]. Nonetheless, SBM algorithms such as Hill Climbing and Simulated Annealing suffer from two major disadvantages. First, they often converge towards local optima and second they can be very sensitive to the initial solution [1].

The PBM methods have been equally explored in feature selection. Different from SBM, PBM represents an iterative improvement in a population of solutions that works as follows. Firstly, the population is initialized. Then, a new population of solutions is generated. Next, the new population is integrated into the existing one by using some selection procedures. The search process is terminated when a certain criterion is satisfied. The most prominent and oldest population-based solution used in feature selection is Genetic Algorithm (GA) [5, 15, 16]. The major roles in GA are the crossover and mutation operations used to couple solutions and to arbitrarily adjust the individual content, to boost diversity aiming to decrease the risk of sticking in local optima.

Another PBM algorithm is the Ant Colony Optimization (ACO), which takes form as a multiagent system, whereby the building unit of this system represents virtual ants as inspired by the behavior of real ants. In nature, a chemical trace called pheromone is left on the ground and is used to guide a group of ants heading for the target point since ants are not able to see very well [6, 17, 18]. Another nature-inspired algorithm is the Particle Swarm Optimization (PSO) algorithm that simulates the social behavior of natural creatures such as bird flocking and fish schooling to discover a place with adequate food [7, 19]. Scatter Search (SS) is another PBM method that recombines solutions elected from a reference set to generate other solutions by building an initial population satisfactory to the criteria of quality and diversity [20].

The next step in feature selection is evaluating the feature subset produced. The evaluation methods can be broadly classified into three categories. First, the filter approach or independent approach evaluates candidate solutions by depending on intrinsic characteristics of the features themselves, without considering any mining algorithm. Filter approach includes several types such as distance [21], information [22], dependency [23], or consistency [24]. Second, the wrapper approach or dependent approach requires one predetermined learning model and selects features with the purpose of improving the generalization performance of that particular learning model [13]. Although the wrapper approach is known to outperform the filter approach with regard to prediction accuracy [25], the method is time-consuming. Third, the embedded approach in feature evaluation attempts to capitalize on advantages of both approaches by implementing the diverse evaluation criteria in different search phases [26]. By integrating the two approaches at different phases, the embedded approach is capable to achieve accuracy of a wrapper approach at the speed of a filter approach. Choosing an evaluation method for particular search method is a critical mission because the interaction between the evaluation method and the search strategy will affect the overall quality of solution.

## 3. Naive Bayes Algorithm

Naive Bayes (NB) algorithm is one of the most effective and efficient inductive learning algorithms for data mining along with machine learning. This algorithm belongs to the wrapper approach. NB is considered a simple classifier based on the classical statistical theory “Bayes theorem.” The Bayesian algorithm is branded “naïve” because it is founded on Bayes Rule, which has a strong supposition that the features are conditionally independent from each other with regard to the class [27]. In the literature, the NB algorithm has proven its effectiveness in various domains such as text classification [28], improving search engine quality [29], image processing [30, 31], fault prediction [32], and medical diagnoses [8].

Naive Bayes classifier works as follows: let  *X*  be a vector of random variables denoting the observed attribute values in the training set  *X* = [*x*_1_, *x*_2_,…, *x*_*n*_]  to certain class label  *c*  in the training set. The probability of each class given the vector of observed values for the predictive attributes can be computed using the following formula:

(1) $$\begin{array}{c} P\left(Y_{j}\;|\;X\right)=\frac{P\left(Y_{j}\right)P\left(X\;|\;Y_{j}\right)}{\sum_{i=1}^{c}P\left(Y_{i}\right)P\left(X\;|\;Y_{i}\right)},\;j=1,\ldots ,c, \end{array}$$

where  *P*(*Y*_*i*_)  is the prior probability of class  *Y*_*i*_ and  *P*(*Y*_*j*_ | *X*)  is the class conditional probability density functions. Basically put, the conditional independence assumption assumes that each variable in the dataset is conditionally independent of the other. This is simple to compute for test cases and to estimate from training data as follows:

(2) $$\begin{array}{c} P\left(X\;|\;Y_{j}\right)=\prod_{i=1}^{n}P\left(X_{i}\;|\;Y_{j}\right),\;j=1,\ldots ,c, \end{array}$$

where *X*_*i*_ is the value of the *i*th attribute in *X* and  *n*  is the number of attributes. Let  *k* be the number of classes, and  *c*_*i*_  is the *i*th class; the probability distribution over the set of features is calculated using the following equation:

(3) $$\begin{array}{c} P\left(x\right)=\prod_{i=1}^{k}p\left(c_{i}\right)p\left(X\;|\;c_{i}\right). \end{array}$$

Effectiveness of Naive Bayes algorithm in classification and learning is attributed to several characteristics such as the following [27].

- High computational efficiency as compared to other wrapper methods because it is inexpensive since it is considered linear time complexity classifier.
- Low variance due to less searching.
- Incremental learning because NB functions work from approximation of low-order probabilities that are deduced from the training data. Hence, these can be quickly updated as new training data are obtained.
- High capability to handle noise in the dataset.
- High capability to handle missing values in the dataset.

Furthermore, NB implementation has no required adjusting parameters or domain knowledge. The major drawback of NB only lies in the assumption of features independence [33]. Despite this, NB often delivers competitive classification accuracy and is widely applied in practice especially as benchmark results. Good survey on the variety of adaptations to NB in the literature can be found in [33].

## 4. Bat Algorithm

The idea of the Bat Algorithm (BA) is to mimic the behaviors of bats when catching their prey. BA was first presented in [34] and it was found to outperform Particle Swarm Optimization (PSO) and Genetic Algorithms (GA) in evaluation using benchmark functions. BA has also been successfully applied to tough optimization problems such as motor wheel optimization problem [35], clustering problem [36], global engineering optimization, and constrained optimization tasks [37–40]. Very recently, two versions of bat-inspired algorithms have been proposed for feature selection [41, 42]. The implementation of BA is more complicated than many other meta-heuristic algorithms [43] because each agent (bat) is assigned a set of interacting parameters such as position, velocity, pulse rate, loudness, and frequencies. This interaction affects the quality of a solution and the time needed to obtain such solution.

The principle of bat algorithm is as follows. A swarm of bats is assumed to fly randomly with velocity *V*_*i*_ at position *X*_*i*_ with a fixed frequency  *f*, varying wavelength  *λ*, and loudness  *A*_0_  to search for a prey. They have the capability to adjust the wavelength of their emitted pulses and regulate the rate of pulse emission  *r* ∈ [0, 1], which is important to determine their closeness of the target. Although the loudness can be different in many ways, the loudness differs from a large (positive)  *A*_0_  to a minimum constant value  *A*_min⁡_. The frequency  *f*  is in the range  [*f*_min⁡_, *f*_max⁡_] that corresponds to a range of wavelengths [*λ*_min⁡_, *λ*_max⁡_]. For example, a frequency range of [20 kHz, 500 kHz] corresponds to a range of wavelengths from 0.7 mm to 17 mm.

## 5. Proposed Naive Bayes-Guided Bat Algorithm 

### 5.1. Frequency

Frequency in the proposed algorithm is represented as a real number as defined in (4). The choice of minimum and maximum frequency depends on the application domain, where *β* is a random number range between 0 and 1. Frequency also affects the velocity as shown in (5). Consider the following:

(4) $$\begin{array}{c} f_{i}=f_{\min}+\left(f_{\max}-f_{\min}\right)\beta . \end{array}$$

### 5.2. Velocity

The velocity of each bat is represented as a positive integer number. Velocity suggests the number of bat attributes that should change at a certain moment of time. The bats communicate with each other through the global best solution and move towards the global best position (solution). The following equation shows the formula for velocity:

(5) $$\begin{array}{c} v_{i}^{t}=v_{i}^{t-1}+\left(x_{*}-x_{i}^{t}\right)f_{i}, \end{array}$$

where  (*x*_∗_ − *x*_*i*_^*t*^)  refers to the difference between the length of global best bat and the length of the *i*th bat. When the difference is positive, this means that the global best bat has more features than those of the *i*th bat. When the result is summed with the previous velocity, it will accelerate the *i*th bat towards the global best bat. If the difference is negative, this means that the *i*th bat has more features than those of the global best bat. Therefore, when the output is summed with the previous velocity, it will decrease the velocity of *i*th bat and help to attract it closer to global best bat. In the proposed Bat Algorithm-Naive Bayes (BANB) algorithm, the maximum velocity was setting (*V*_max⁡_) equal to (1/3)∗*N*, where  *N*  is the number of features. In the proposed BANB, (2) is used to adjust the velocity during each iteration; therefore, the proposed algorithm is adaptive for feature selection problem in order to mimic the original algorithm behavior. Velocity representation is also one major difference between BANB and the Binary Bat Algorithm (BBA) [42]. In BBA, the velocity is calculated for each single feature; hence, the algorithm is more time-consuming and departing from the original algorithm attitude. On the contrary, the velocity in the proposed BANB is calculated once for the entire solution; hence, the velocity amount determines the piece of change.

### 5.3. Position Adjustment

In the proposed algorithm, each bat position is formulated as a binary string of length *N*, where  *N*  is the total number of features. Each feature is represented by bit, where “1” means that the corresponding feature is selected and the “0” means that it is not selected. The positions are categorized into two groups according to the bit difference between the *i*th bat and the global best bat in order to align exploitation and exploration during searching.

The bat's position is adjusted depending on one of the following conditions. In the case where the velocity of *i*th bat is lower or equal to the number of different bits, *i*th bat will copy some features from global best bat, thus moving towards global best bat, while still exploring new search space. In the case where the velocities of *i*th bat are higher than the velocity of global best bat, then the *i*th bat will import all features from the global best bat to be the same as the global best bat with a few different bits to facilitate further exploitation. The following equation shows the position adjustment, where  *x*  is bat position, and  *v*  is the velocity of the *i*th bat at time *t*:

(6) $$\begin{array}{c} x_{i}^{t}=x_{i}^{t-1}+v_{i}^{t}. \end{array}$$

### 5.4. Loudness

Loudness *A*_*i*_ in the proposed algorithm is represented as the change in number of features at certain time during local search around the global best bat, as well as local search around the *i*th bat. The formula for loudness is shown in (7), where  *A*_*i*_^*t*^  is the average loudness of all the bats at certain iteration and  *ε* ∈ [−1,1]. The value for sound loudness (*A*) ranges between the maximum loudness and minimum loudness. Consider the following:

(7) $$\begin{array}{c} x_{\text{new}}=x_{\text{old}}+\varepsilon A^{t}. \end{array}$$

Generally, the loudness value will decrease when the bat starts approaching the best solution. The following equation shows that the amount of decrease is determined by *α*:

(8) $$\begin{array}{c} A_{i}^{t+1}=\alpha A_{i}^{t}. \end{array}$$

The value for sound loudness also plays an important role in obtaining good quality solutions within a reasonable amount of time. The choice of the maximum and minimum loudness depends on the domain of application and also the size of the dataset. In the proposed BANB algorithm, the maximum loudness has been determined empirically as (1/5)∗*N*, where *N*  is number of features. Value for maximum loudness is dynamic depending on number of features in certain dataset. For example, when  *A*_max⁡_ = 3  and  *A*_min⁡_ = 1, the bat begins to reduce the number of features from 3 features to 2 features and the value then becomes a single feature when it gets closer to the target.

### 5.5. Pulse Rate

Pulse rate *r*_*i*_ has the role to decide whether a local search around the global best bat solution should be skipped or otherwise. Higher pulse rate will reduce the probability of conducting a local search around the global best and vice versa. Therefore, when the bat approaches the best solution, pulse rate value will increase and subsequently reduce the chances to conduct a local search around the global best. The amount of increase is determined by  *γ*  as defined in the following:

(9) $$\begin{array}{c} r_{i}^{t+1}=r_{i}^{0}\left[1-\exp\left(-\gamma t\right)\right]. \end{array}$$

### 5.6. Fitness Function

Each candidate solution is using a fitness function defined in (10), where  *P*(*Y*_*J*_ | *X*)  is the classification accuracy, TF is the total number of all features, and SF is the number of selected features.  *δ*  and *φ* are two parameters corresponding to the weight of classification accuracy and subset length, where  *δ* ∈ [0, 1]  and *φ* = 1 − *δ*. From (10), we can see that the importance of classification accuracy and subset size is weighted differently. Generally, classification accuracy is given more weight than the size of the subset. In this experiment, the two parameters have been set as follows:  *δ* = 0.9, *φ* = 0.1. Consider the following:

(10) $$\begin{array}{c} {\text{Sol}}_{A}=\delta \cdot P\left(Y_{j}\;|\;X\right)+\varphi \cdot \frac{\text{TF}-\text{SF}}{\text{TF}}. \end{array}$$

The complete algorithm for the proposed hybrid BA guided by Naive Bayes classifier (BANB) is shown in Algorithm 1.

## 6. Experiments and Results

The objective of the experiments is to evaluate the performance of the proposed Naive Bayes-guided Bat Algorithm (BANB) in terms of number of features selected and the classification accuracy achieved. To achieve this objective, we compared the number of features and classification accuracies of BANB with several well-known algorithms, which are Genetic Algorithms (GA) [44], Particle Swarm Optimization (PSO) [45], and Geometric Particle Swarm Optimization (GPSO) [46]. Similar to the proposed BANB, we also used Naive Bayes classifier for all comparative algorithms as the attribute evaluator. However, the parameters for the algorithms had the same settings as those used by the original authors. For the proposed algorithm, the parameters were set to the following values: population size = 25 and decrease sound loudness and increase pulse rate both are set to 0.6. The initial value of pulse rate is equal to 0.2. The proposed BANB algorithm and other algorithms were run for 20 times with different initial solutions. Following [4, 17], all the algorithms were terminated after 250 iterations.

### 6.1. Description of Dataset

For the experiments, twelve datasets were considered to cover both cases of binary and multiclass data. Three of the datasets, namely, M-of-N, Exactly, and Exactly2, were sourced from [47]. M-of-N is an artificial binary class since the decision attribute consisting of two class labels and the dataset were generated from a uniform distribution to create the artificial domain. Exactly and Exactly2 are artificial binary classification datasets, generated based on x-of-y concepts, which are not linearly separable and are known to be difficult for many classes of learning algorithms [47]. The remaining datasets were taken from the UCI data repository [48]. The datasets are Vote, Credit, LED, Derm, Derm2, Lung, WQ, Heart, and Mushroom. Vote is widely used as a binary classification dataset in the literature. The dataset represents votes for each of the U.S. House of Representatives congressmen with the class label democrat and republican. Credit dataset is a binary classification data that is concerned with credit card applications. LED dataset in display domain is a multiclass classification data as the class label includes ten possible values in which the first seven features determine the class label of a pattern, whilst the rest of the 17 features are irrelevant.

Derm and Derm2 represent real data in dermatology concerning differential diagnosis of erythematosquamous diseases. The class labels contain six values, which refer to six different diseases. Lung dataset is the pathological types of Lung cancer that aims to demonstrate the power of the optimal discriminant plane even in ill-posed settings [49]. WQ is a multiclass label dataset that originated from the daily measures of sensors in an urban waste water treatment plant. The idea is to categorize the operational state of the plant with the purpose of predicting faults out of the state variables of the plant at each of the phases in the water treatment procedure. Heart is a binary class data that contains 76 attributes although all the published experiments reference to using only 14 of the original attributes. This data has been used to predict heart diseases, whereby the class label of zero and one refers to the absence or existence of heart disease in the patient. Finally, Mushroom is a binary class dataset that includes characterization of hypothetical samples identical to 23 types of gilled mushrooms in the *Agaricus* and *Lepiota* family.  Table 1 shows the characteristics of the datasets.

### 6.2. Results for Feature Selection Experiment

In this experiment, we compared the proposed BANB against GA [44], PSO [45], and GPSO [46] in terms of the number of features selected from the original dataset.  Table 2 provides the comparison results. The number of features obtained from the comparative algorithms in Table 2, and the best results are highlighted in bold. Then the results are statistically tested using two tests, Kolmogorov-Smirnov and Levene test [50]. However, the Kolmogorov-Smirnov and Levene test did not meet the assumptions of normality distribution and equality of variance which then led us to use Wilcoxon test. Essentially, this test is an alternative to the paired *t*-test, when the assumption of normality or equality of variance is not met [51]. Wilcoxon test is rated to be a robust estimate tool that depended on the rank estimation [52].  Table 3 presents Wilcoxon test results for the proposed BANB algorithm against other feature selection algorithms. From Table 3, between the brackets refer to the algorithm that performs better than another algorithm. The results of Wilcoxon test are considered to be statistically significant at  *P*  less than 0.05 and are highly significant at  *P*  less than 0.01.

### 6.3. Results for Classification Accuracy Experiment

The second part of the experiment was to evaluate and compare the average classification accuracies achieved by BANB and other comparative algorithms over 10 runs, using 10-fold cross-validation method. Three well-known classifiers were employed for the purpose of evaluating the resulting subsets among different classifiers, which were JRip PART and J48 [53]. Tables 4, 5, and 6 show the average classification accuracy and standard deviation values from the experiment.

## 7. Discussions

In selecting the feature subset, Table 2 shows that the proposed BANB algorithm obtained the smallest number of features across all datasets except for LED.  Table 3 confirmed that the difference between BANB and the remaining comparative algorithms is highly significant except for LED and M-of-N datasets. More significantly, BANB is able to reduce the number of features up to a single feature in five datasets as shown in Table 2. In evaluating the feature subset, if we take into consideration the interaction between classification accuracy and number of features selected by the proposed BANB algorithm as compared to other algorithms, we can categorize the results into three cases. In the first case, a reduced number of features deliver the same classification accuracy. This is shown in the Exactly dataset that produced similar classification accuracy in both JRip and J48 classifiers and even higher accuracy in PART classifier. On the contrary, features selected by other algorithms included more features, which indicate that some of the features selected are redundant. This can be seen clearly in the Exactly2 dataset when all solutions achieved exactly the same accuracy in spite of variance in the number of selected features.

In the second case, the proposed algorithm reduced the number of features while at the same time increased the classification accuracy. For example, BANB selected only two features from the Lung dataset as opposed to additional eight features among other algorithms. The difference between the numbers of features selected is attributed to noisy features, which cause a decrease in classification accuracy such as in the Vote dataset. In the third case, smaller feature subset that is selected delivers a slightly lower classification accuracy, such as in Heart and Mushroom dataset with the exception of LED dataset. All algorithms could deliver the same accuracy with the same number of features because the LED dataset contains very protruding features.

Finally, it can be noted from Tables 4, 5, and 6 that the classification accuracies achieved by the proposed BANB algorithm are in less disagreement or very close across three different classifiers. This can be noted obviously from the experimental results using Exactly, Credit, Lung, and Derm datasets. To support this finding, we calculated standard deviation for each dataset over the three different classifiers and we averaged the values for each algorithm. The results were as follows: BANB equals 0.36, GPSO equals 0.99, PSO equals 1.04, and, finally, GA equals 1.11. This implies that the proposed feature selection algorithm BANB has better generalization as compared to other feature selection algorithms. Results from Table 2 also show that BANB is capable of selecting the same number of features for 9 out of 12 datasets over 20 iterations. This is followed by GA, GPSO, and, finally, PSO. It can be noted that the standard deviation values in Tables 4, 5, and 6 are zeros for 9 datasets. This means that our proposed BANB could obtain certain number of features with exactly the same features for each iteration. As a consequence, BANB showed the highest stability among all comparative algorithms.

## 8. Conclusion

In this paper, a new hybrid feature selection algorithm has been presented. The Bat Algorithm employed Naïve Bayes Algorithm to intelligently select the most convenient feature that could maximize the classification accuracy while ignoring redundant and noisy features. We compared our proposed algorithm with three other algorithms using twelve well-known UCI datasets. The performance was evaluated from four perspectives, which are the number of features, classification accuracy, stability, and generalization. From the experiments, we can conclude that the proposed Naïve Bayes-guided Bat Algorithm (BANB) outperformed other meta-heuristic algorithms with a selection of feature subsets that are significantly smaller with a less number of features. In terms of classification accuracy, BANB has proven to achieve equal, if not better results as compared to other algorithms. For stability, the proposed algorithm is more stable than other algorithms. Finally, from the perspective of generalization of results, the resulting features produced by BANB are also more general than other algorithms in practice. For future work, further investigations are required to observe the behavior of the proposed algorithm in gene expression and very high-dimensional datasets.

## Figures and Tables

**Algorithm 1:**
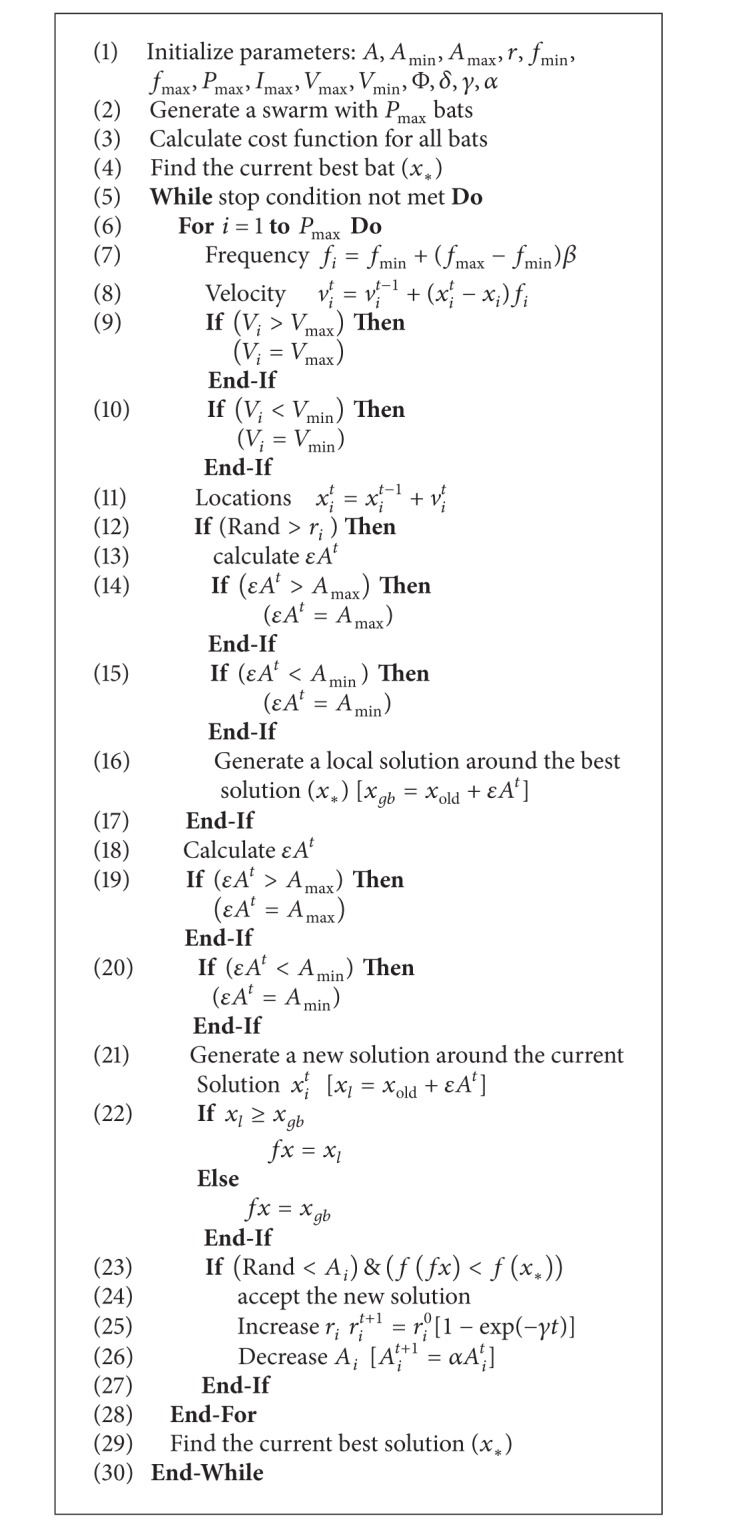
BANB Algorithm.

**Table 1:** Dataset characteristics.

Datasets	No. of features	No. of samples
Lung	56	32
WQ	38	521
Derm2	34	358
Derm	34	366
LED	24	2000
Mushroom	22	8124
Credit	20	1000
Vote	16	300
Heart	13	294
Exactly2	13	1000
Exactly	13	1000
M-of-N	13	1000

**Table 2:** Average of selected features.

Datasets	BANB	GA	GPSO	PSO
M-of-N	**6**	8	6.9	7.6
Exactly	**1**	7	7	6.9
Exactly2	**1**	3	3	2.9
Heart	**4**	6	5.7	6.25
Vote	**1**	3	3.1	3.55
Credit	**1**	10	11.7	11.9
Mushroom	**1**	5.75	6.15	5.9
LED	**5**	**5**	**5**	**5**
Derm	**12.3**	18.1	17.1	21.5
Derm2	**11.45**	17.75	17.3	20.75
WQ	**5.9**	20.1	20.15	21.05
Lung	**2**	8.6	7.95	8.7

**Table 3:** Wilcoxon test results.

	Wilcoxon test		
	GA-BANB	GPSO-BANB	PSO-BANB
M-of-N	.000 (BANB )	.013 (BANB )	.013 (BANB )
Exactly	.000 (BANB )	.000 (BANB )	.000 (BANB )
Exactly2	.000 (BANB )	.000 (BANB )	.000 (BANB )
Heart	.000 (BANB )	.000 (BANB )	.000 (BANB )
Vote	.000 (BANB )	.000 (BANB )	.000 (BANB )
Credit	.000 (BANB )	.000 (BANB )	.000 (BANB )
Mushroom	.000 (BANB )	.000 (BANB )	.000 (BANB )
LED	1	1	1
Derm	.000 (BANB )	.000 (BANB )	.000 (BANB )
Derm2	.000 (BANB )	.000 (BANB )	.000 (BANB )
WQ	.000 (BANB )	.000 (BANB )	.000 (BANB )
Lung	.000 (BANB )	.000 (BANB )	.000 (BANB )

**Table 4:** Average classification accuracy with standard deviation for JRip.

Datasets	JRip			
	BANB	GA	GPSO	PSO
M-of-N	98.9 ± 0	99.1 ± 0	97.62 ± 4.32	98.92 ± 0.23
Exactly	68.8 ± 0	68 ± 0	68 ± 0	68.01 ± 0.22
Exactly2	75.8 ± 0	75.8 ± 0	75.8 ± 0	75.8 ± 0
Heart	80.61 ± 0	81.97 ± 0	81.66 ± 0.49	82.47 ± 5.33
Vote	95 ± 0	93.66 ± 0	93.92 ± 0.43	94.42 ± 0.35
Credit	71.6 ± 0	70.7 ± 0	70.75 ± 0.75	70.48 ± 0.79
Mushroom	98.52 ± 0	100 ± 0	99.46 ± 0.48	99.33 ± 0.45
LED	100 ± 0	100 ± 0	100 ± 0	100 ± 0
Derm	93.30 ± 1.69	89.39 ± 0.90	90.18 ± 2.41	91.08 ± 0.78
Derm2	90.77 ± 1.77	89.64 ± 1.64	90.47 ± 1.31	89.35 ± 1
WQ	70.99 ± 1.57	69.49 ± 1.35	68.59 ± 1.61	68.34 ± 1.39
Lung	87.5 ± 0	84.68 ± 1.77	83.74 ± 4.37	84.05 ± 2.73

**Table 5:** Average classification accuracy with standard deviation for PART.

Datasets	PART			
	BANB	GA	GPSO	PSO
M-of-N	100 ± 0	100 ± 0	98.53 ± 4.62	100 ± 0
Exactly	68.8 ± 0	65.6 ± 0	65.6 ± 0	65.93 ± 0.82
Exactly2	75.8 ± 0	75.8 ± 0	75.8 ± 0	75.8 ± 0
Heart	79.59 ± 0	80.27 ± 0	80.57 ± 0.49	79.79 ± 1.44
Vote	94.33 ± 0	94.33 ± 0	94.42 ± 0.15	94.49 ± 0.36
Credit	71.7 ± 0	72.9 ± 0	72.24 ± 1.14	71.81 ± 1.05
Mushroom	98.52 ± 0	100 ± 0	99.33 ± 0.46	99.39 ± 0.41
LED	100 ± 0	100 ± 0	100 ± 0	100 ± 0
Derm	94.39 ± 1.76	94.73 ± 0.86	95.65 ± 0.99	95.62 ± 0.71
Derm2	93.15 ± 2.80	95.19 ± 2.82	95.27 ± 0.76	95.61 ± 0.76
WQ	68 ± 0.7	67.76 ± 1.78	68.70 ± 1.28	67.07 ± 2.92
Lung	78.12 ± 0	80.93 ± 4.52	81.21 ± 5.84	80.27 ± 4.36

**Table 6:** Average classification accuracy and standard deviation for J48.

Datasets	J48			
	BANB	GA	GPSO	PSO
M-of-N	100 ± 0	100 ± 0	98.53 ± 4.64	100 ± 0
Exactly	68.8 ± 0	68.8 ± 0	68.8 ± 0	68.8 ± 0
Exactly2	75.8 ± 0	75.8 ± 0	75.8 ± 0	75.8 ± 0
Heart	79.93 ± 0	79.25 ± 0	79.25 ± 0	79.08 ± 0.28
Vote	95 ± 0	94 ± 0	94.13 ± 0.23	94.39 ± 0.30
Credit	71.7 ± 0	72.2 ± 0	72.21 ± 0.46	72.08 ± 1.08
Mushroom	98.52 ± 0	100 ± 0	99.49 ± 0.45	99.39 ± 0.41
LED	100 ± 0	100 ± 0	100 ± 0	100 ± 0
Derm	93.92 ± 1.04	95.02 ± 0.52	94.64 ± 0.77	94.36 ± 0.68
Derm2	91.25 ± 2.44	94.38 ± 1.67	93.73 ± 0.51	94.32 ± 0.89
WQ	65.08 ± 0.46	69.11 ± 1.35	68.30 ± 2.01	68.32 ± 2.82
Lung	87.5 ± 0	79.37 ± 4.70	80.62 ± 6.21	82.18 ± 4.67
